# Supplementary material for: Functional role of TRIM E3 ligase oligomerization and regulation of catalytic activity
Source: EMBO J. 2016 May 6;35(11):1204–18. doi: 10.15252/embj.201593741 (PMC4864278; doi:10.15252/embj.201593741)
Supplement: Supplementary file 1 — Expanded View Figures PDF [file EMBJ-35-1204-s001.pdf]

Expanded View Figures

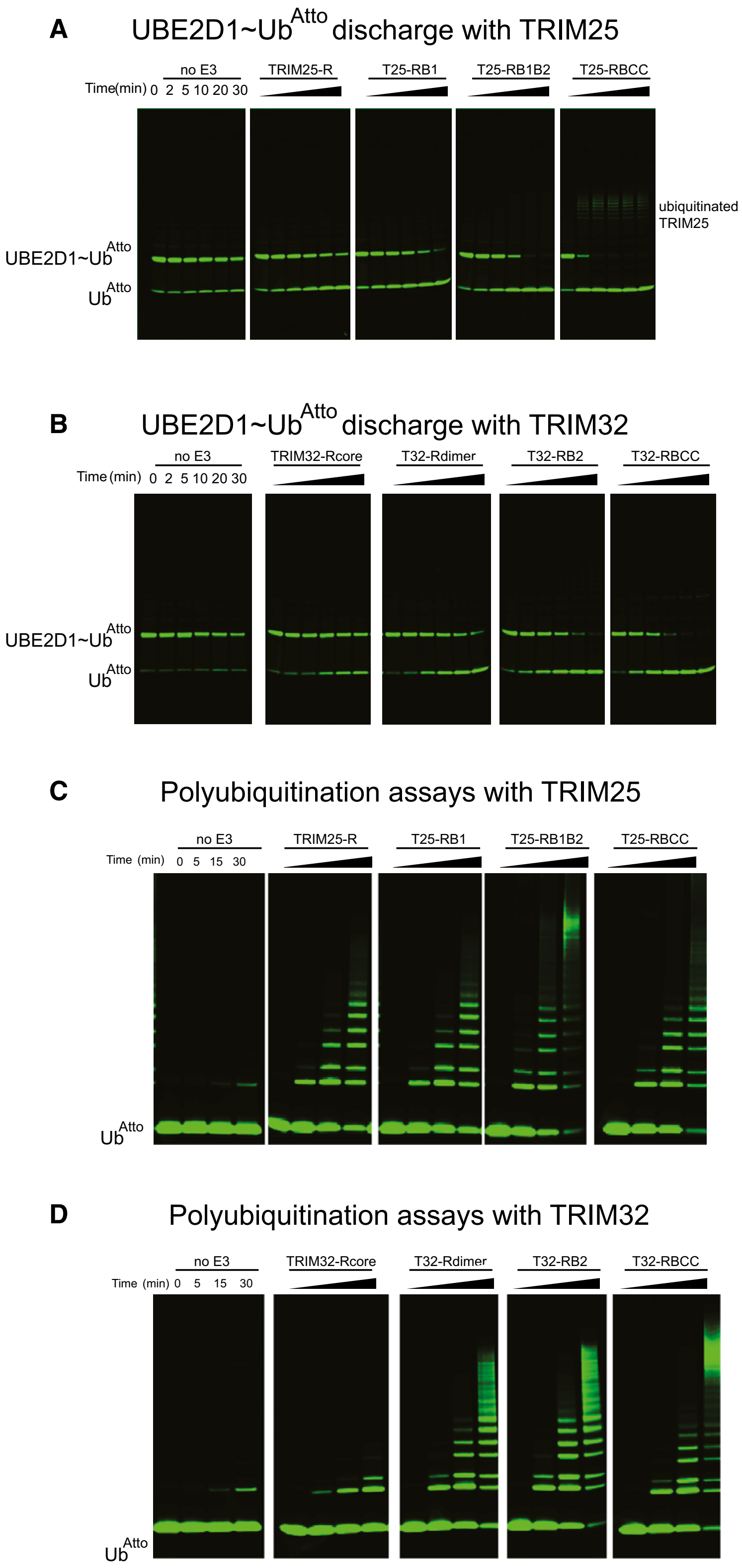

Figure EV1. E2~Ub discharge and poly-ubiquitination assays using a fluorescently labelled ubiquitin Ub^{Atto}.

A Representative gels of the discharge assays with UBE2D1~Ub^{Atto} and different TRIM25 constructs. The gels were scanned with a Storm 869 Scanner and the bands for free Ub^{Atto} integrated.

B Representative gels of the discharge assays with UBE2D1~Ub^{Atto} and different TRIM32 constructs. The gels were scanned with a Storm 869 Scanner and the bands for free Ub^{Atto} integrated.

C Poly-ubiquitination assays using UBE2N/UBE2V1 and different TRIM25 constructs supplemented with fluorescent Ub^{Atto}.

D Poly-ubiquitination assays using UBE2N/UBE2V1 and different TRIM32 constructs supplemented with fluorescent Ub^{Atto}.

 © 2016 Francis Crick Institute

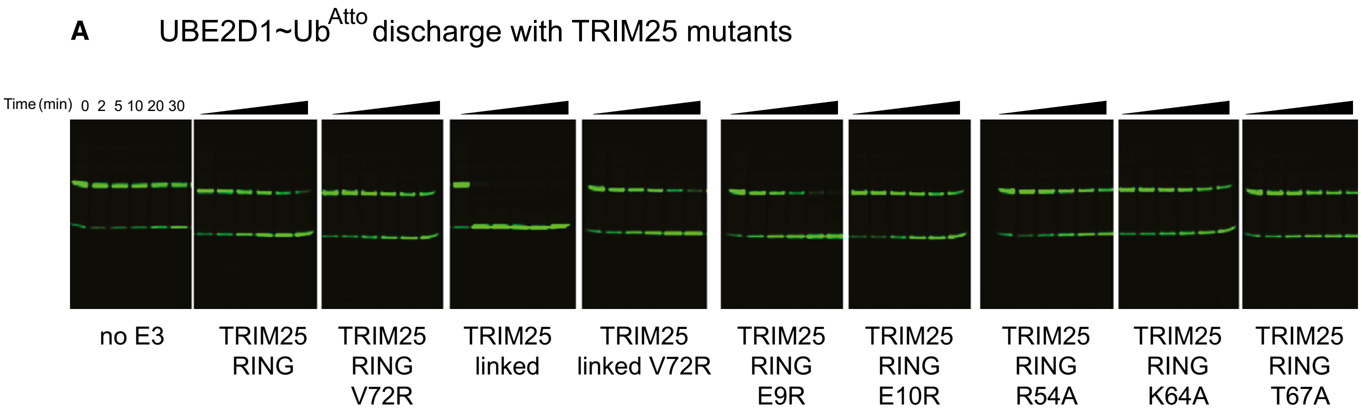

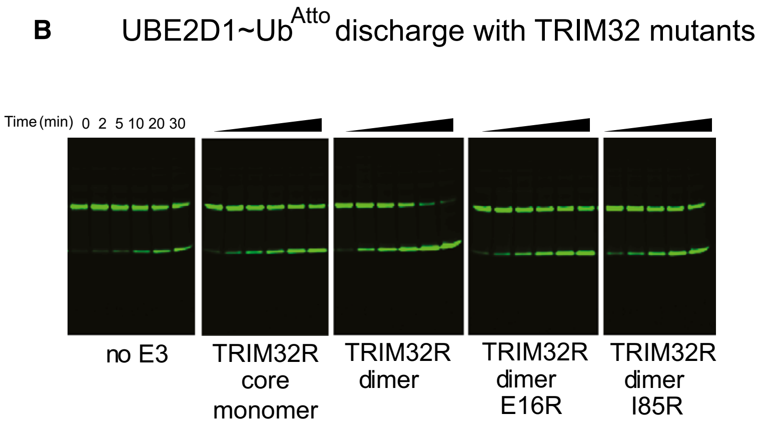

Figure EV2. UBE2D1~Ub^{Atto} discharge assays with TRIM25 and TRIM32 RING mutants.

A Representative gels of the discharge assays with UBE2D1~Ub^{Atto} and different TRIM25 RING mutants. The gels were scanned with a Storm 869 Scanner and the bands for free Ub^{Atto} integrated.

B Representative gels for the equivalent assays with TRIM32 RING mutants.

© 2016 Francis Crick Institute

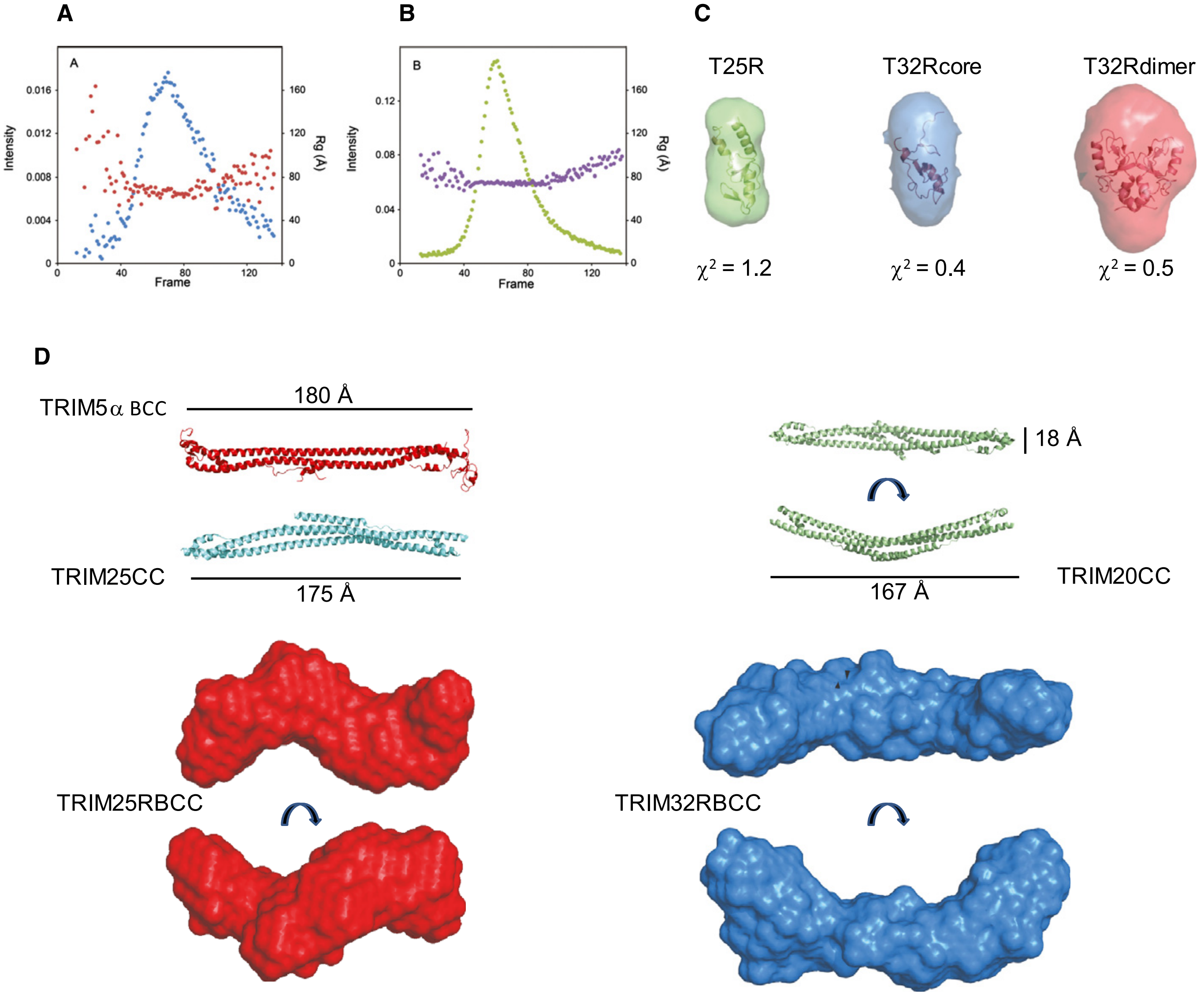

Figure EV3. SAXS data recording and modelling of TRIM25 and TRIM32.

A, B Size-exclusion chromatography in line with SAXS data recording for TRIM25 RBCC (A) and TRIM32 RBCC (B) at the SWING beamline at SOLEIL. The intensity (blue and green) and Rg (red and purple) profiles are reported as a function of the frames recorded at equal time intervals.

C *Ab initio* low-resolution envelopes calculated with the program DAMAVER for TRIM25 RING (green), TRIM32 RING core (blue) and TRIM32 RING dimer superimposed to their structures by the program SUPCOMB. The values of the chi-square are calculated by the program CRYSOL.

D Low-resolution envelopes calculated by DAMAVER for TRIM25 RBCC (red) and TRIM32 RBCC (blue) in different orientations presented in scale with the structures of TRIM5α BCC (PDB 4TN3), TRIM25 CC (PDB 4LTB) and TRIM20 CC (PDB 4CG4).

 © 2016 Francis Crick Institute

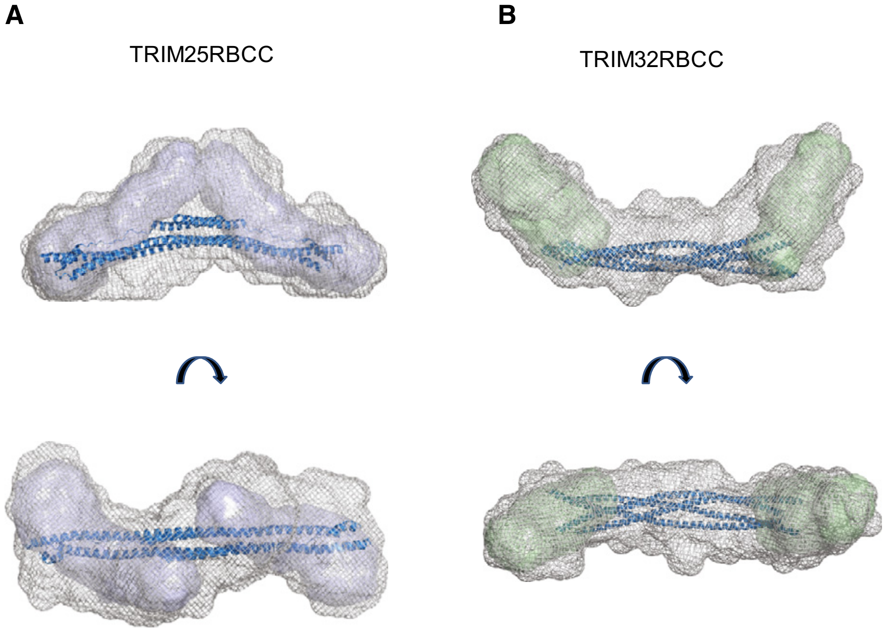

Figure EV4. Models for the domain architecture of TRIM25 and TRIM32 RBCC.

A, B The internal volume of the low-resolution average envelopes of TRIM25 (A) and TRIM32 (B) RBCC, obtained from the SAXS analysis, can accommodate the two monomeric TRIM25 RB1B2 envelopes (represented in blue, see Fig 6) and the two dimeric TRIM32 RB2 envelopes (in green, see Fig 6) at the N-terminus on each side of the central coiled-coil spanning the length of the molecule. The structure coordinates of TRIM25 CC and TRIM20 CC were used to model the central coiled-coil region of TRIM25 RBCC and TRIM32 RBCC, respectively. For TRIM32RBCC, 2 copies of the same coiled-coil coordinates (rotated of 180° relative to the log helical axis) were used.

© 2016 Francis Crick Institute
