# Supplementary material for: Functional role of TRIM E3 ligase oligomerization and regulation of catalytic activity
Source: EMBO J. 2016 May 6;35(11):1204–18. doi: 10.15252/embj.201593741 (PMC4864278; doi:10.15252/embj.201593741)

Figure 2: original InstantBlue stained gels

A-TRIM25

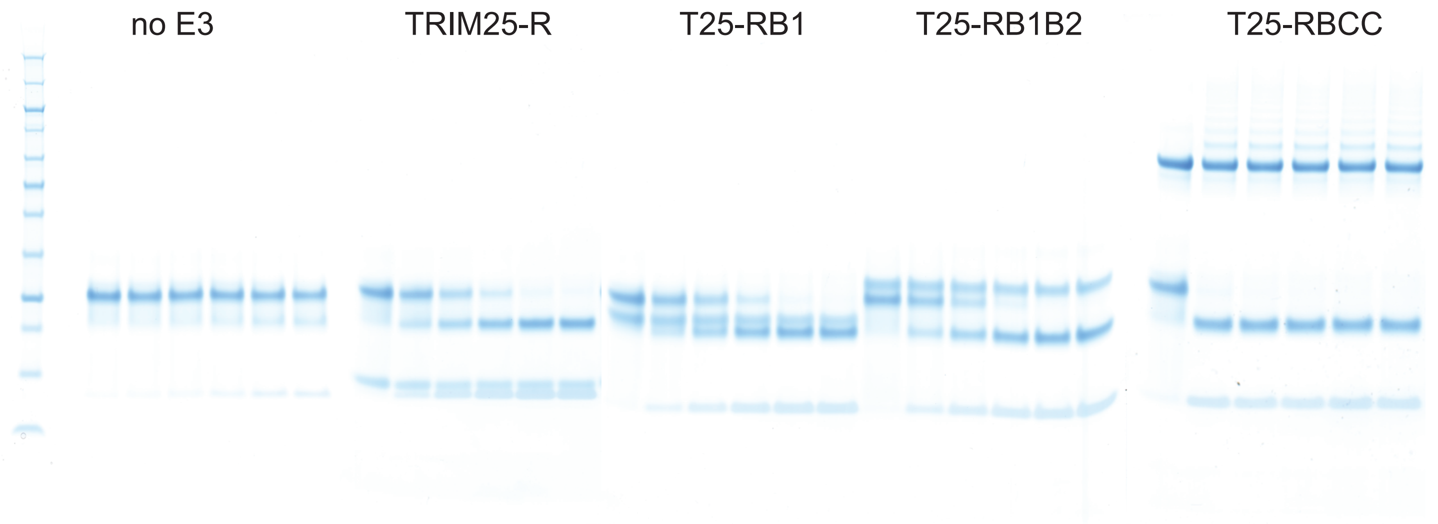

C-TRIM32

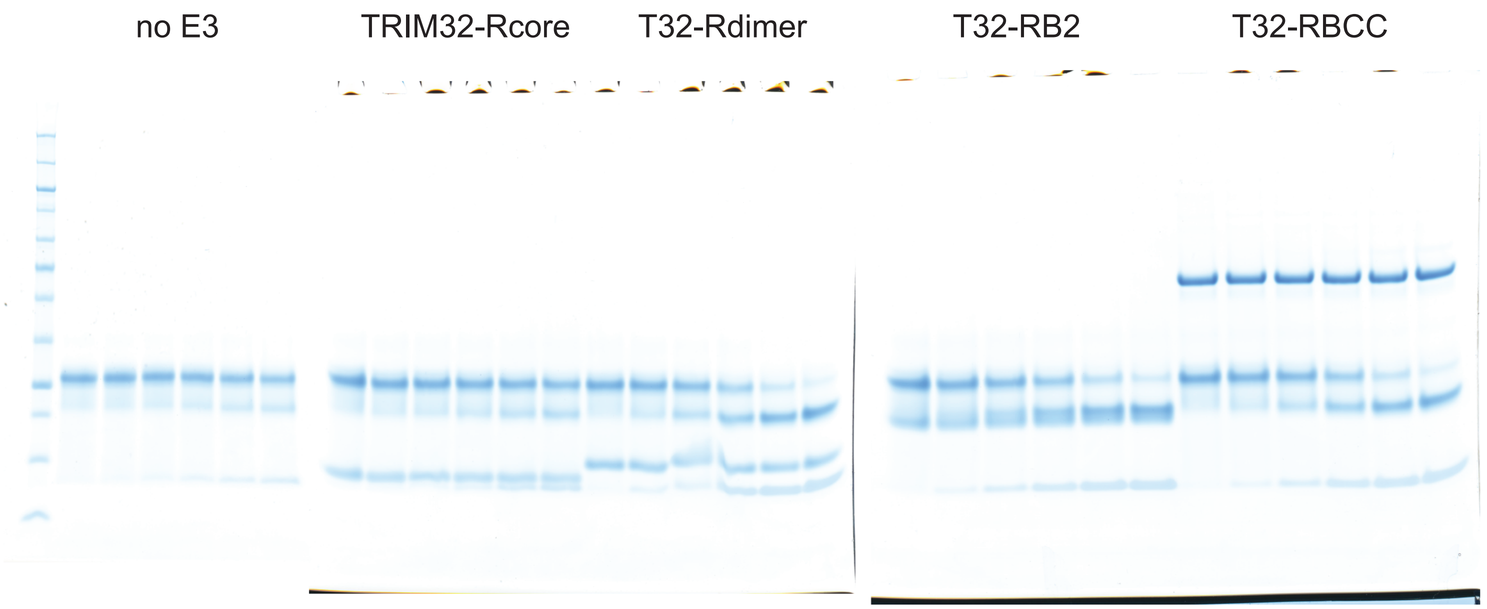

Figure 2: original InstantBlue stained gels

E-TRIM25

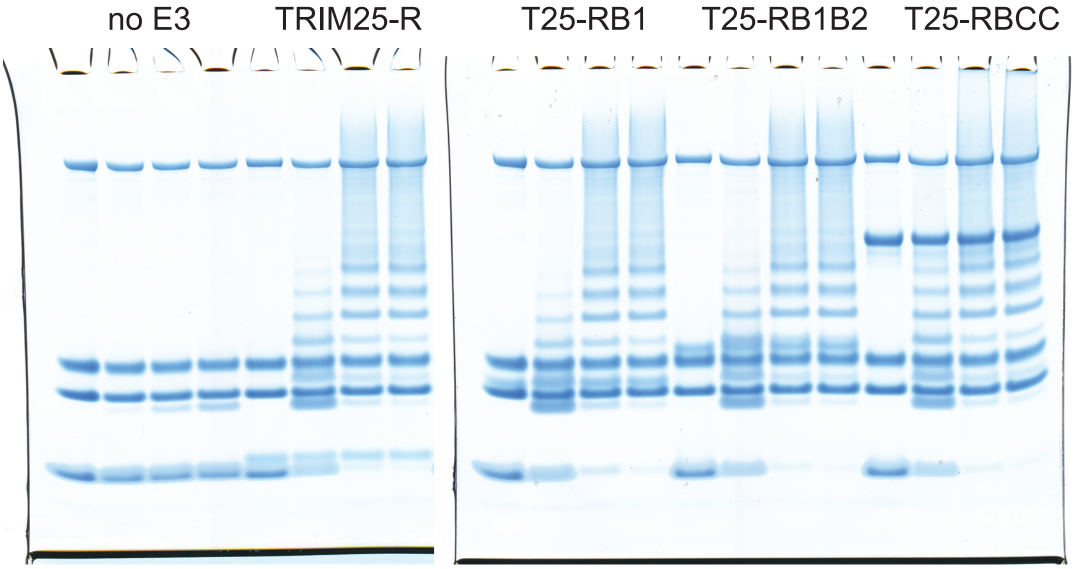

G-TRIM32

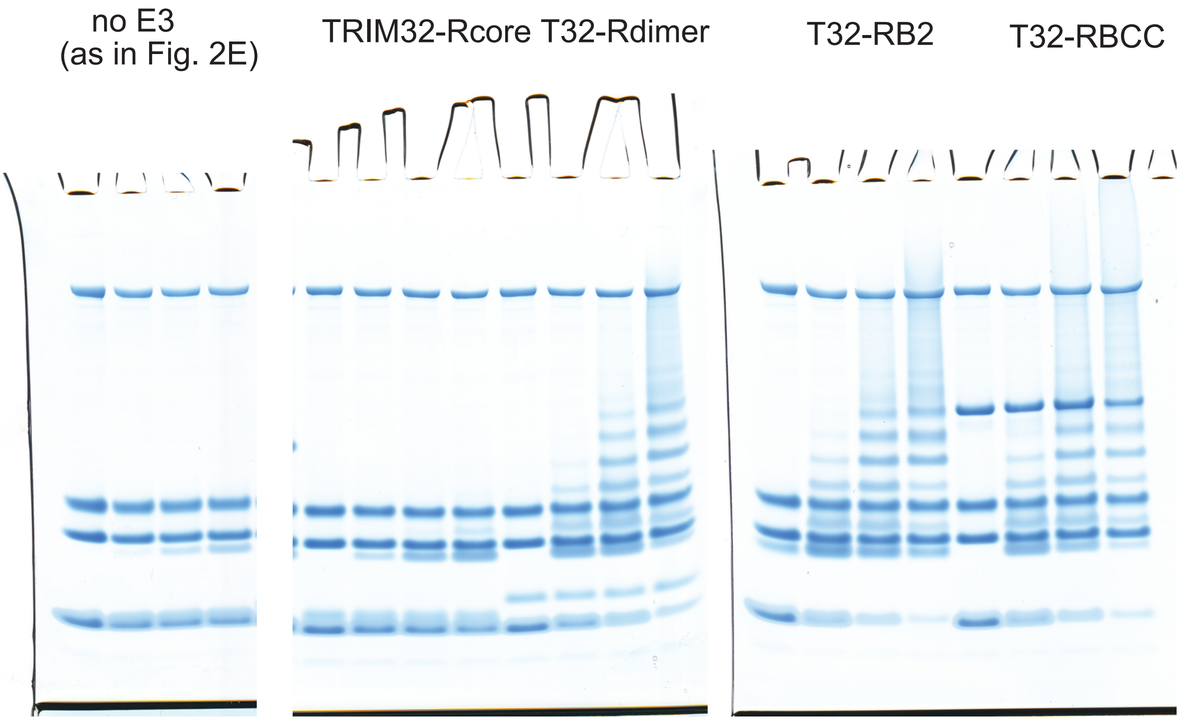

Supplement: Supplementary file 3 — Source Data for Figure 2 [file EMBJ-35-1204-s002.pdf]
