# Supplementary figures and images for: Functional role of TRIM E3 ligase oligomerization and regulation of catalytic activity
Source: EMBO J. 2016 May 6;35(11):1204–18. doi: 10.15252/embj.201593741 (PMC4864278; doi:10.15252/embj.201593741)

Figure 4: original InstantBlue stained gels

C

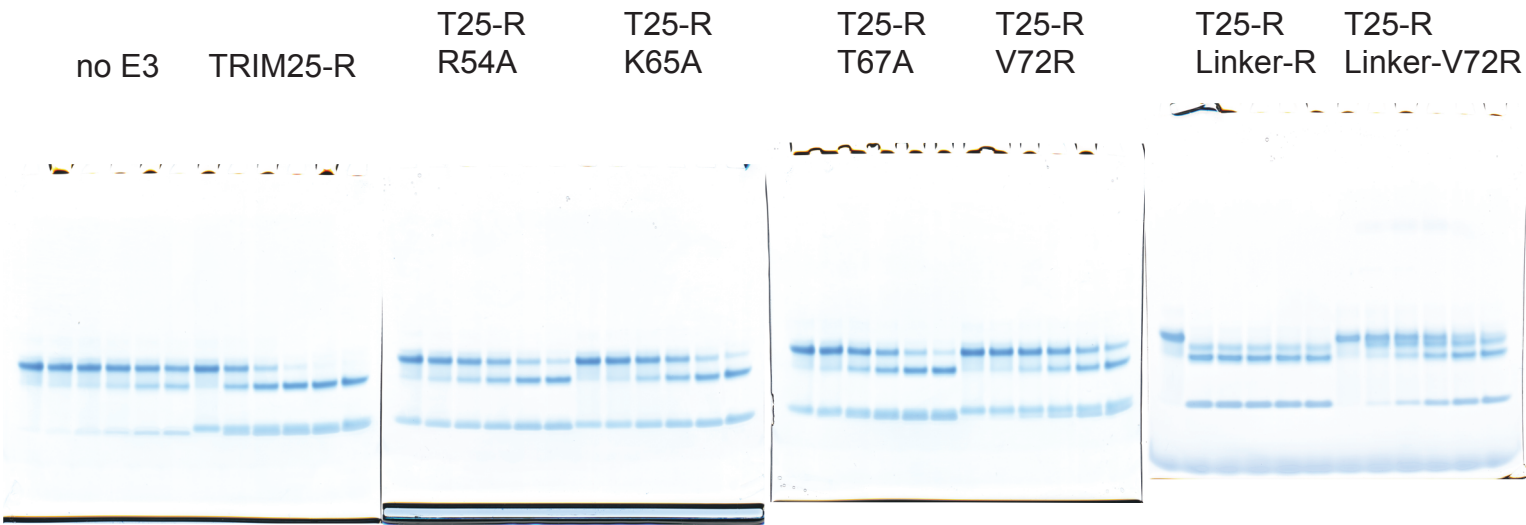

D

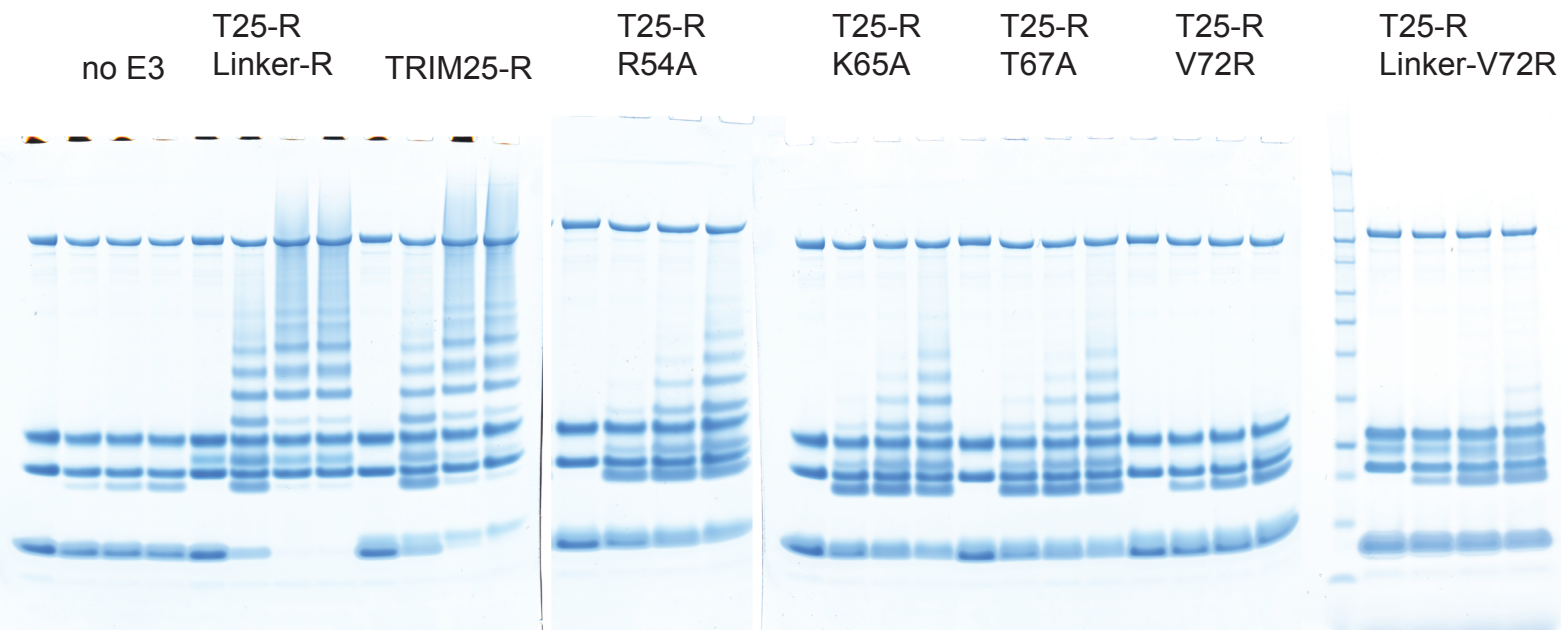

Supplement: Supplementary file 4 — Source Data for Figure 4 [file EMBJ-35-1204-s003.pdf]

Figure 5: original InstantBlue stained gels

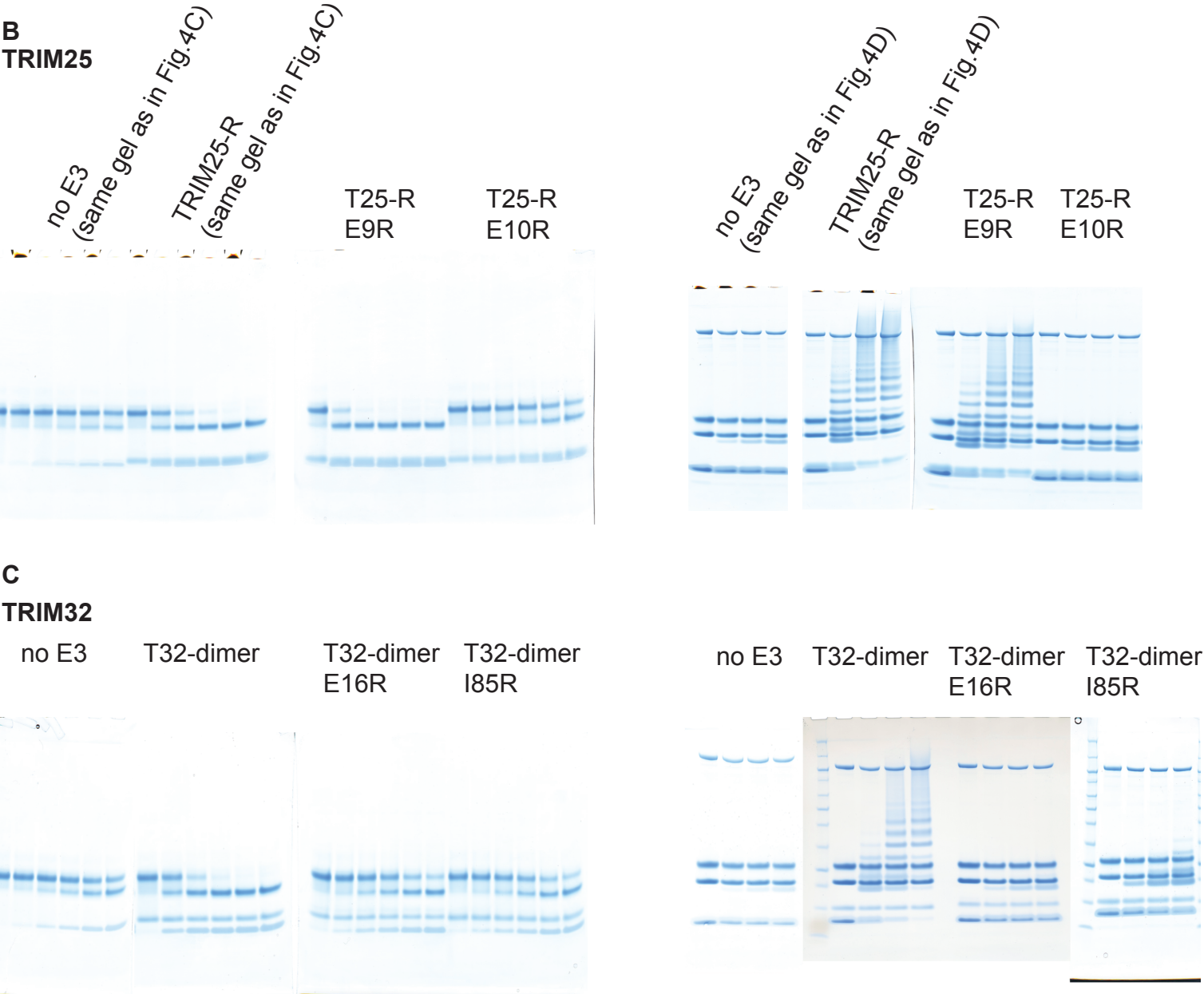

Supplement: Supplementary file 5 — Source Data for Figure 5 [file EMBJ-35-1204-s004.pdf]
